# Comparative efficacy of oral administrated afoxolaner (NexGard™) and fluralaner (Bravecto™) with topically applied permethrin/imidacloprid (Advantix^®^) against transmission of *Ehrlichia canis* by infected *Rhipicephalus sanguineus* ticks to dogs

**DOI:** 10.1186/s13071-016-1636-9

**Published:** 2016-06-17

**Authors:** Frans Jongejan, Dionne Crafford, Heidi Erasmus, Josephus J. Fourie, Bettina Schunack

**Affiliations:** Utrecht Centre for Tick-borne Diseases (UCTD), FAO Reference Centre for Ticks and Tick-borne Diseases, Faculty of Veterinary Medicine, Utrecht University, Yalelaan 1, 3584 CL Utrecht, The Netherlands; Department of Veterinary Tropical Diseases, Faculty of Veterinary Science, University of Pretoria, Private Bag X04, Onderstepoort, 0110 South Africa; Clinvet International (Pty) Ltd, Uitzich Road, Bainsvlei, Bloemfontein South Africa; Zoology Department, University of Johannesburg, Corner of Kingsway and University Roads, Aucklandpark, South Africa; Bayer Animal Health GmbH, Monheim, Germany

**Keywords:** Permethrin/imidacloprid, Afoxolaner, Fluralaner, *Ehrlichia canis*, *Rhipicephalus sanguineus* ticks, Transmission blocking, Speed of kill

## Abstract

**Background:**

The ability of the topical spot-on Advantix^®^ (50 % permethrin/10 % imidacloprid) to prevent transmission of *Ehrlichia canis* by infected *Rhipicephalus sanguineus* ticks to dogs has previously been reported. The recent market introduction of chewable tablets containing the novel compounds, afoxolaner (NexGard™) and fluralaner (Bravecto™) enabled us to conduct a comparative efficacy study with respect to the ability of these three products to block transmission of *E. canis* by ticks to dogs. The speed of kill, immediate drop-off rate and anti-attachment efficacy of the respective products were also studied.

**Methods:**

The study was a blinded parallel group design, wherein 32 dogs were randomised into four different groups of eight dogs. Group 1 served as negative placebo control, group 2 and 3 were treated on Days 0, 28 and 56 with NexGard™ and Advantix^®^, respectively. Group 4 was dosed once on Day 0 with Bravecto™. For tick efficacy assessments 50 non-infected ticks were placed onto the dogs on Days 30, 35, 42, 49, 56, 63, 70, 77 and 84 and on animal tick counts were performed at 3 h, 6 h and 12 h after infestation. To evaluate the ability to block transmission of *E. canis*, each dog was challenged by releasing 80 adult *E. canis*-infected *R. sanguineus* ticks into their sleeping kennels on Days 31, 38, 45 and 52. The animals were monitored for clinical signs of monocytic ehrlichiosis (pyrexia and thrombocytopenia) and were tested for *E. canis* DNA by PCR and for specific antibodies using IFA. A dog was considered infected with *E. canis* if both PCR and IFA yielded positive test results up to Day 84.

**Results:**

Mean arithmetic tick counts on dogs treated with the Advantix^®^ spot-on were significantly (*P* < 0.0005) lower throughout the study as compared with the negative controls and was, with respect to the speed of kill and resulting onset of acaricidal efficacy, superior over NexGard™ and Bravecto™ at all time points in the 12 h period observed (3 h, 6 h and 12 h). None of the dogs treated with the Advantix^®^ spot-on became infected with *E. canis*, whereas six out of eight untreated control dogs acquired the infection. Furthermore, *E. canis* infection was diagnosed in four out of eight dogs treated with NexGard™ and in two out of eight dogs treated with Bravecto™.

**Conclusions:**

The speed of kill of the two recently registered systemic compounds against *R. sanguineus* was not sufficiently fast to prevent transmission of *E. canis* and resulted in only low partial blocking and protection capacity while Advantix^®^ effectively blocked transmission of *E. canis* to dogs in the challenge period and thus provided adequate protection for dogs against monocytic ehrlichiosis.

## Background

Ectoparasites are no longer considered just a nuisance, but are recognized as important vectors of disease agents causing a range of vector-borne diseases (VBDs), with ticks especially transmitting a great variety of infectious organisms [1]. Thus evaluating the value of tick control products should not be limited to their acaricidal efficacy as such, but also in the light of their ability to prevent tick-borne diseases (TBDs).

*Rhipicephalus sanguineus* (Latreille, 1806), the brown dog tick, has a worldwide distribution between latitudes 50° N and 30° S, where it is predominantly encountered on domestic dogs [2]. Recently, it has been recognized that *R. sanguineus* may actually consist of several different taxonomic entities and that currently *R. sanguineus* (*sensu stricto*) cannot be satisfactorily assigned to any particular population of ticks because the type specimen has not been properly preserved as a reference [3]. Despite this, the epithet *R. sanguineus* is used here when referring to brown dog ticks utilised in the present study.

The main importance of brown dog ticks is related to their capacity to transmit a broad range of bacterial and protozoan pathogens, such as *Rickettsia conorii*, *Anaplasma platys* (transmission suspected but not confirmed), *Babesia vogeli* and *Hepatozoon canis* [1]. The most important bacterium, however, is *Ehrlichia canis*, the causative agent of canine monocytic ehrlichiosis (CME), which distribution coincides with the distribution of the vector tick and affects the health of dogs all over the world [4, 5]. *Ehrlichia canis* is a gram-negative intra-cellular bacterium with a preference for the cytoplasm of circulating canine monocytes. The symptomatology that ensues have been defined as CME and include various clinical signs. Clinical signs of CME, as well as laboratory findings associated with this condition, may include lethargy, fever, anorexia, enlarged lymph nodes, pancytopenia (in particular thrombocytopenia), epistaxis [6] and the clinical presentation can be classified as either acute, subacute or a more chronic form [6, 7]. Doxycycline is the drug of choice for the treatment of CME [6, 7].

Brown dog ticks are three-host ticks with all stages adapted to dogs and as a result this tick can built up large population densities sustained by dogs in their kennels and associated human dwellings, where they also can pose a threat to human health [8]. For example, it was only a decade ago that the transmission of Rocky Mountain Spotted Fever to humans in the USA could be attributed to human-biting *R. sanguineus* ticks, which are usually considered to be strictly host-specific with all stages feeding on dogs [9]. *Rhipicephalus sanguineus* can complete several cycles per year in (sub) tropical regions of the world, whereas in the more temperature regions there are usually discrete peaks of adult tick activity in spring and summer, whereas in autumn immature stages emerge on the same dog population. This is for instance seen in the Mediterranean region [10].

Hence, effective acaricidal control of *R. sanguineus* is important in order to avoid the build-up of large numbers of ticks as well as the prevention of chronic or fatal canine monocytic ehrlichiosis.

A key issue with respect to any acaricidal product is its capacity to prevent infection by blocking transmission of pathogens that can be transmitted by ticks and may cause disease in companion animals. This capacity depends on the time it takes the product to be fully effective, but also on the time interval before the tick is able to transmit the pathogen [11].

Comparatively little information is available regarding the actual speed of transmission of tick-borne pathogens. The time range for pathogen transmission varies broadly, from immediate transmission e.g. for the TBE (tick-borne encephalitis) virus [11] to transmission only after one to two days of attachment e.g. for *Babesia canis* [12]. For rickettsial pathogens the transmission time is somewhat vaguely given with four to 48 h [13]. However recent studies into specific rickettsials showed transmission to happen earlier than previously thought. The transmission of *E. canis* by *R. sanguineus* ticks was shown to start within a few hours after attachment, with dogs already infected three hours after exposure to ticks [14]. For *Rickettsia rickettsii* transmission by *Amblyomma aureolatum* a transmission time of > 10 h is shown for unfed ticks, reduced to a minimum of 10 min after attachment for ticks that had already fed [15].

Recently, clinical laboratory models have been successfully developed to assess the capacity of ectoparasiticides for their ability to prevent, forestall or completely block the transmission of pathogens by ticks and fleas and are additionally used to define transmission times more precisely [16].

The first model was established for the transmission of *Babesia canis* by infected *Dermacentor reticulatus* ticks and examined the ability to block transmission for a combination of fipronil, amitraz and (s)-methoprene [17]. This model was consequently used to determine the blocking capacities of a 4.5 % flumethrin and 10 % imidacloprid collar formulation [18] and lately for afoxolaner and fluralaner containing systemic products [19, 20].

Thereafter, a model for transmission of *E. canis* by infected *R. sanguineus* ticks was established again first to examine the blocking capacity of a combination of fipronil, amitraz and (S)-methoprene [21]. This model was subsequently also used to determine the blocking capacity of a permethrin/imidacloprid spot-on formulation [14], a 4.5 % flumethrin and 10 % imidacloprid collar formulation [22], and recently, a novel combination of fipronil and permethrin [23].

Recently, a novel class of orally applied systemic products was introduced, whose mode of action requires ticks to attach to the host and commence feeding to become effective. For both systemic compounds published studies demonstrated the ability to effectively block transmission of *B. canis* by *D. reticulatus* ticks [19, 20]. However a number of studies comparing speed of kill between these novel systemic actives and topically applied products with local activity and repellent properties found significant differences in speed of kill within the first 12 h of attachment [24, 25]. These results raise the question if these novel systemic actives have the capacity to block transmission of quicker transmitted pathogens.

In the study reported in this paper, we assessed the capacity to block transmission of *E. canis* by infected *R. sanguineus* ticks to dogs for the two recently approved systemic compounds: afoxolaner (NexGard™, Merial Limited, Lyon, France) [26] and fluralaner (Bravecto™, MSD Animal Health Innovation GmbH, Schwabenheim, Germany) [27] in comparison to a reference topical 50 % permethrin/10 % imidacloprid spot-on formulation (Advantix^®^, Bayer Animal Health, Leverkusen, Germany), which had already shown its ability to block transmission of *E. canis* in the first month of application [14]. The speed of kill, immediate drop-off rate and anti-attachment efficacy of the respective products was also studied.

## Methods

### Ethical approval and study outline

This study was conducted in compliance with the Good Clinical Practice (GCP) guideline (Veterinary International Conference on Harmonization GL9) [28] and the European Medicines Agency guidelines regarding testing of anti-parasitic substances for treatment and prevention of tick and flea infestation in dogs and cats [29]. Ethical approval for conduct of the study was obtained from the “Clinvet Committee for Animal Ethics and Welfare” ethics body prior to conduct of the study.

The study was performed using purpose-bred dogs belonging to Clinvet, at the Clinvet study site near Bloemfontein, Free State, South Africa. The study was randomised, blinded and employed a parallel group design. Inclusion criteria for dogs into the study were: clinically healthy, older than six months, not clinically pregnant, not treated with any ectoparasiticide for at least 12 weeks prior to the start of the study, sero-negative for *E. canis* by immunofluorescence assay (IFA) and negative for *E. canis* deoxyribonucleic acid (DNA) by polymerase chain reaction (PCR). Thirty-two purpose-bred beagles and mongrels belonging to Clinvet that complied with these inclusion criteria were divided into weight ranges (< 10 kg; > 10 kg to 20 kg; and > 20 kg). Health status at inclusion was confirmed by a veterinarian during clinical examination. Dogs were ranked within sex (14 males and 18 females) in descending order of individual live tick counts prior to veterinary product or placebo administration, and subsequently blocked into eight blocks of four dogs each. Within blocks, dogs were randomly allocated to four groups using Microsoft Excel software. A non-blinded person randomly assigned the groups to four coded groups using the same software package. Blinded personnel only had access to group codes and not group numbers. The study was thus conducted on four groups of eight dogs each. All dogs, identifiable by a microchip number, were individually housed in tick-proof kennels and observed daily throughout the duration of the study. In order to eliminate possible bias, additionally to the use of a placebo spot-on product, persons involved in the post-treatment observations were different from those that performed group allocations and treatments.

The study was laid out over a timeframe of three months (84 days), with the actual challenge periods in month two (Days 28 to 56) for *E. canis* transmission blocking and months two and three (Days 28 to 84) for tick efficacy assessments.

### Treatments and rescue treatments

**Fig. 1:**
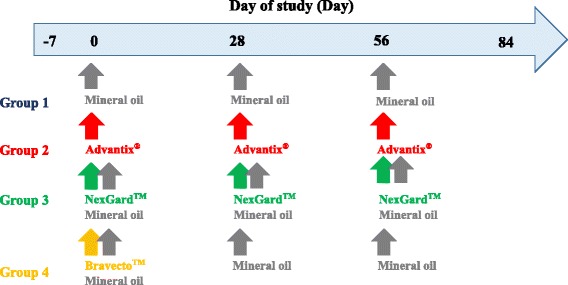
Treatment time point layout

Dogs allocated to group 1 served as negative control and received a placebo spot-on compound (mineral oil), dogs in group 2 were treated with Advantix^®^ spot-on solution for dogs (50 % permethrin/10 % imidacloprid), those in group 3 received NexGard™ chewable tablets for dogs (afoxolaner) and those in group 4 received Bravecto™ chewable tablets for dogs (fluralaner). Treatment regime is shown in Fig. 1 including placebo compound administrations for orally treated groups. Bravecto™ chewable tablets were administered on Day 0 only, based on the up to three month efficacy against ticks registered label claim of this product. All products were administered within weight classes as per label instructions. The placebo treatment applied to dogs in the Control group as well as the orally treated NexGard™ and Bravecto™ groups consisted of mineral oil only and was applied in three or four spots along the midline of the back. In the Advantix^®^-treated group between 10.42 mg/kg and 24.51 mg/kg imidacloprid and between 52.08 mg/kg and 122.55 mg/kg permethrin were applied by parting the hair and applying the product directly onto the skin in three or four spots along the midline of the back. In the NexGard™-treated group between 2.54 mg/kg and 5.48 mg/kg afoxolaner were administered orally. In the Bravecto™-treated group between 25.25 mg/kg and 47.62 mg/kg fluralaner were administered orally.

Animals were screened weekly using PCR and IFA. A positive PCR result provided confirmation of clinical diagnosis and hence the necessity to perform rescue treatment. Animals that tested negative (both PCR and IFA) on the last day of the study were not rescue-treated. Six animals in control group 1, no animals in Advantix^®^ group 2, four animals in NexGard™ group 3 and two animals in Bravecto™ group 4 were rescue-treated with a commercial product containing doxycycline (Doxydog 100 mg and 50 mg, registration numbers G2636 and G2688 respectively) at the recommended dose rates and treatment regime.

### Tick efficacy assessments

Efficacy assessments focused on early time points after tick infestation as defined in the following sections:

#### Tick infestations for efficacy assessments

**Fig. 2:**
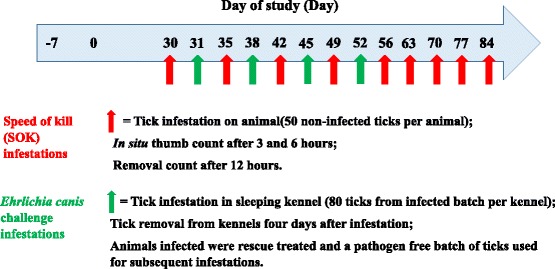
Tick challenge time point layout

A laboratory-bred strain of pathogen-free *R. sanguineus* (European origin, French strain) was used for artificial infestations. Each dog was infested with 50 ticks on days indicated in Fig. 2. Dogs (not sedated) were placed in an infestation crate, followed by the placement of 50 ticks on the dogs. The dogs were subsequently restrained inside the crates for 10 min before closing the mesh cover to confine the animal in the crate for a period of 12 h. Ticks, which dropped off the dogs during the first 10 min, were not placed back, unless shaken off by the dog.

#### Tick counting procedures

The tick counting procedures were designed to allow calculation of speed of kill, immediate drop-off and anti-attachment efficacy as described in the sections to follow, which in turn aided in the interpretation of efficacy in preventing *E. canis* transmission. On animal tick counts for speed of kill assessment at 3 h, 6 h and 12 h after infestation were performed on Days 30, 35, 42, 49, 56, 63, 70, 77 and 84. *In situ* thumb counts were performed 3 h (± 15 min) and 6 h (± 30 min) after each infestation. During these *in situ* counts, sexes were not distinguished, but ticks were categorised as live or dead. Calculation was performed according to the most current guidelines [30] and as a result engorgement status was not considered during efficacy calculations. Tick removal counts were performed 12 h (± 30 min) after each infestation. During removal counts ticks were counted within sex (male or females) and same general status as defined for the *in situ* counts. On the days and time points specified for *in situ* counts above, the ticks that dropped off the dogs were collected from the infestation crates. Collection took place during the time the dog was removed from the infestation crate for tick counts.

### Methods for calculating efficacy and comparing groups

All efficacy results reported were based on arithmetic means as requested by current guidelines [30].

The ***speed of kill efficacy*** was calculated as the acaricidal efficacies [30] for the treated groups at the different assessment time points (3 h and 6 h *in situ* and 12 h removal counts). Speed of kill efficacy calculations were based on arithmetic mean tick counts using Abbott’s formula:

$$ \mathrm{Speed}\ \mathrm{of}\ \mathrm{kill}\ \mathrm{efficacy}\ \left(\%\right) = 100\kern0.5em \times \kern0.5em \left(\mathrm{M}\mathrm{c}\hbox{--} \mathrm{M}\mathrm{t}\right)/\mathrm{M}\mathrm{c} $$

where Mc = Arithmetic mean number of live ticks on dogs in the control group at a specific time point and Mt = Arithmetic mean number of live ticks on dogs in the respective treated groups at a specific time point.

The ***immediate tick drop-off rate*** was calculated based on the number of ticks recovered off the animal (i.e. free in the infestation crate) within 3 h of infestation as follows:

$$ \mathrm{Immediate}\ \mathrm{drop}\hbox{-} \mathrm{off}\ \mathrm{rate}\ \left(\%\right)=\left[\left(50\hbox{--} \mathrm{M}\mathrm{c}\right)\hbox{--} \left(50\hbox{--} \mathrm{M}\mathrm{t}\right)\right]/\left(50\hbox{--} \mathrm{M}\mathrm{c}\right)\times 100 $$

where Mc = Arithmetic mean number of total ticks collected off animal in the control group at the 3 h time point and Mt = Arithmetic mean number of total ticks collected off animal in the respective treated groups at the 3 h time point.

The ***anti-attachment efficacies*** at 6 h and 12 h post-infestation were calculated based on attached tick counts on dogs only. The aim was to evaluate if ticks that remained on the animals at 3 h after infestation actually attached to the animals by 6 and 12 h, respectively as follows:

$$ \mathrm{Anti}\hbox{-} \mathrm{attachment}\ \mathrm{efficacy}\ \left(\%\right)=100\kern0.5em \times \kern0.5em \left(\mathrm{T}\mathrm{m}\mathrm{c}\hbox{--} \mathrm{T}\mathrm{m}\mathrm{t}\right)/\mathrm{T}\mathrm{m}\mathrm{c} $$

where Tmc = Total attached (arithmetic mean of live and dead) ticks on the dogs in the control group at the respective time point, and Tmt = Total attached (arithmetic mean of live and dead) ticks on the dogs in the respective treated groups at the respective time point.

The groups were compared using an ANOVA after a logarithmic transformation on the tick (count + 1) data. The proportion of animals in each group was also compared. SAS Version 9.3 TS Level 1 M2 was used for all the statistical analyses. The level of significance was set at 5 %; all tests were two-sided.

### Tick infestations to assess *Ehrlichia canis* blocking efficacy and monitoring of infection

Ticks used in this study derived from the same laboratory-bred strain of *R. sanguineus* used for the acaricidal efficacy determination and were artificially infected with a South African strain of *E. canis* using methods previously published [16, 31]. In order to simulate environmental tick challenges, ticks were released into the sleeping kennels of dogs on days indicated in Fig. 2. Ticks used were unfed, at least two weeks old and had a balanced sex ratio. The average infection rate of the tick batch used was 3.8 % and 80 ticks were released into each kennel to ensure an adequate environmental challenge.

Following environmental challenges ticks were removed after four days on Days 35, 42, 49 and 56. Moreover, each sleeping kennel was visually inspected for detached ticks, which were removed and the kennel cleaned.

Infection with *E. canis* was monitored by clinical examinations, rectal temperature records, platelet counts, as well as by testing blood samples by PCR and IFA.

All animals were observed daily for general health. Also clinical examinations (all dogs) were performed during acclimatisation for inclusion purposes, as well as on Days -1, 27, 34, 41, 48, 55, 62, 69, 76 and 83. Rectal body temperatures were recorded at least three times per week from Day 35 to Day 84. When dogs displayed abnormally high body temperatures (> 39.4 °C), a further measurement was taken the following day to evaluate for persistent pyrexia.

Blood specimens were collected for *E. canis* DNA detection by PCR and for platelets counts on Days -8, 30, 35, 42, 49, 56, 63, 70, 77 and 84. As PCR target, a specific fragment of the *dsb* gene of *E. canis* was amplified according to conditions previously published [23]. Conventional PCR was employed for detection of *E. canis* in animal blood and quantitative real-time PCR was employed for parasite load detection. Platelets counts were conducted by PathCare Veterinary Laboratory. Platelet concentration was evaluated as part of the complete blood count (CBC). The concentration was determined using a laser optic method. Smear examinations were performed on all abnormal platelet concentrations.

Serum was collected on Days -6, 35, 42, 49, 56, 63, 70, 77 and 84 and frozen at -20 °C until assayed for the detection of specific *E. canis* antibodies using a commercial IFA test kit (Megascreen^®^ Fluoehrlichia c. test kit manufactured by MegaCor Diagnostik, Hörbranz, Austria). IgG titres of 1:40 and greater were considered to reflect infection (i.e. positive result).

Additional clinical examinations to that specified on the before mention days were conducted on any animal that displayed signs associated with ehrlichiosis. These signs included, but were not limited to, persistent pyrexia, thrombocytopenia and lethargy. For all animals with suspected ehrlichiosis, additional blood specimens for PCR were collected as needed to confirm the diagnosis.

Whilst a positive PCR result provided confirmation of clinical diagnosis and hence the necessity to perform rescue treatment, an efficacy failure (successfully infected with *E. canis* as employed in blocking and risk reduction calculations as described below) was defined as a dog that was found positive for *E. canis* DNA by PCR analysis and also seroconverted (tested positive for *E. canis* antibodies).

### Methods for calculating the *Ehrlichia canis* blocking efficacy

The ***blocking efficacy*** per infected animal considers the number of animals that were successfully infected. Blocking efficacy per infected animal for the treatment group was calculated as follows:

$$ \mathrm{Blocking}\ \mathrm{efficacy}\ \left(\%\right)=100\times \left(\mathrm{T}\mathrm{c}-\mathrm{T}\mathrm{t}\right)/\mathrm{T}\mathrm{c} $$

where Tc = Total number of infected dogs in the negative control group and Tt = Total number of infected dogs in the respective treatment group.

Whilst this is the simplest calculation method, the underlying assumption is that all animals were exposed to the same challenge pressure, which is not always the case.

Navarro et al. (2015) [32] first employed the number of infective challenges in their calculation method, as deduced from one of the result tables. Jongejan et al. (2015) [23] first defined a formula for this calculation method, that considers not solely number of infected animals but calculates the percentage of protection in comparison to the number of infective challenges.

***Percentage of protection*** was defined and calculated as follows:

$$ \mathrm{Protection}\ \left(\%\right)=100\kern0.5em \times \kern0.5em \left(\mathrm{I}\mathrm{c}\mathrm{C}-\mathrm{I}\mathrm{c}\mathrm{T}\right)/\mathrm{I}\mathrm{c}\mathrm{C} $$

where IcC = the “infection proportion” calculated as the number of infected animals in the control group divided by the total number of pre-infection challenges with ticks from a batch infected with *E. canis* in the control group and IcT = the “infection proportion” calculated as the number of infected animals in the respective treatment groups divided by the total number of pre-infection challenges with ticks from a batch infected with *E. canis* in the respective treatment groups.

## Results

No adverse events related to administration of any of the veterinary products assessed occurred during this study.

### On animal tick count results

Mean tick counts on animals in the control group indicated that an adequate infestation was reached on all assessment days and all assessment time points: 3 h (Table 1), 6 h (Table 2) and 12 h (Table 3).

**Table 1:** Mean arithmetic on animal tick counts three hours after infestation of dogs with *Rhipicephalus sanguineus*

Day	Arithmetic mean tick counts 3 hours after infestation^a^												
	Control	Advantix^®^				NexGard™				Bravecto™			
	M	M	*F*	*df*	*P*	M	*F*	*df*	*P*	M	*F*	*df*	*P*
Day 30	49.4	15.6	86.69	3	< 0.0001	49.5	86.69	3	0.9615	49.4	86.69	3	1.0000
Day 35	47.0	14.4	76.37	3	< 0.0001	48.0	76.37	3	0.7100	46.8	76.37	3	0.9258
Day 42	48.1	10.8	184.50	3	< 0.0001	48.3	184.50	3	0.9499	49.5	184.50	3	0.4916
Day 49	47.4	12.0	184.57	3	< 0.0001	46.8	184.57	3	0.7412	49.6	184.57	3	0.2399
Day 56	47.6	16.9	207.73	3	< 0.0001	48.5	207.73	3	0.5693	47.4	207.73	3	0.8705
Day 63	44.4	18.5	33.43	3	< 0.0001	46.4	33.43	3	0.5648	48.4	33.43	3	0.2537
Day 70	46.4	11.3	80.02	3	< 0.0001	46.5	80.02	3	0.9652	48.5	80.02	3	0.4606
Day 77	46.4	13.6	39.97	3	< 0.0001	42.8	39.97	3	0.3063	44.6	39.97	3	0.6188
Day 84	48.6	12.0	311.86	3	< 0.0001	48.6	311.86	3	1.0000	48.3	311.86	3	0.7994

*Abbreviations*: *M* arithmetic mean, *F*, F-statistic, *df*, degrees of freedom, *P*, *P*-value (one-way ANOVA with a treatment effect in comparison with control group)

^a^counts based on ticks attached to the dogs, excluding ticks in the crates

**Table 2:** Mean arithmetic on animal tick counts six hours after infestation of dogs with *Rhipicephalus sanguineus*

Day	Arithmetic mean tick counts 6 hours after infestation^a^												
	Control	Advantix^®^				NexGard™				Bravecto™			
	M	M	*F*	*df*	*P*	M	*F*	*df*	*P*	M	*F*	*df*	*P*
Day 30	20.1	3.3	8.12	3	0.0005	15.5	8.12	3	0.2920	22.9	8.12	3	0.5283
Day 35	35.0	3.4	17.07	3	< 0.0001	27.3	17.07	3	0.1092	26.9	17.07	3	0.0939
Day 42	31.6	2.3	32.17	3	< 0.0001	24.5	32.17	3	0.0366	26.6	32.17	3	0.1346
Day 49	35.4	1.1	27.53	3	< 0.0001	34.6	27.53	3	0.8665	31.1	27.53	3	0.3444
Day 56	33.4	2.6	16.24	3	< 0.0001	29.3	16.24	3	0.4017	26.1	16.24	3	0.1457
Day 63	32.0	6.3	9.30	3	< 0.0001	25.8	9.30	3	0.2447	27.1	9.30	3	0.3619
Day 70	27.6	0.6	34.78	3	< 0.0001	24.6	34.78	3	0.3224	23.4	34.78	3	0.1646
Day 77	25.5	2.6	10.24	3	< 0.0001	23.4	10.24	3	0.6452	18.1	10.24	3	0.1175
Day 84	32.4	1.8	59.82	3	< 0.0001	34.9	59.82	3	0.3882	31.1	59.82	3	0.6646

*Abbreviations*: M, arithmetic mean; *F*, F-statistic; *df*, degrees of freedom; *P*, *P*-value (one-way ANOVA with a treatment effect in comparison with control group)

^a^counts based on ticks attached to the dogs, excluding ticks in the crates

**Table 3:** Mean arithmetic on animal tick counts 12 hours after infestation of dogs with *Rhipicephalus sanguineus*

Day	Arithmetic mean tick counts 12 hours after infestation^a^												
	Control	Advantix^®^				NexGard™				Bravecto™			
	M	M	*F*	*df*	*P*	M	*F*	*df*	*P*	M	*F*	*df*	*P*
Day 30	35.3	3.5	14.56	3	< 0.0001	24.0	14.56	3	0.0288	22.5	14.56	3	0.0143
Day 35	31.8	0.9	15.15	3	< 0.0001	25.6	15.15	3	0.2202	16.3	15.15	3	0.0036
Day 42	35.5	2.3	16.27	3	< 0.0001	30.1	16.27	3	0.3156	15.5	16.27	3	0.0007
Day 49	34.5	1.5	22.62	3	< 0.0001	33.1	22.62	3	0.7640	24.3	22.62	3	0.0318
Day 56	33.6	1.9	16.01	3	< 0.0001	34.9	16.01	3	0.8187	22.0	16.01	3	0.0402
Day 63	28.5	5.8	5.77	3	0.0003	17.3	5.77	3	0.0507	19.5	5.77	3	0.1136
Day 70	26.6	0.3	24.65	3	< 0.0001	20.1	24.65	3	0.0714	25.1	24.65	3	0.6687
Day 77	24.6	3.9	7.68	3	0.0002	24.0	7.68	3	0.9001	19.0	7.68	3	0.2639
Day 84	31.8	0.3	55.67	3	< 0.0001	36.0	55.67	3	0.1986	22.9	55.67	3	0.5283

*Abbreviations*: *M*, arithmetic mean, *F*, F-statistic, *df*, degrees of freedom, *P*, *P*-value (one-way ANOVA with a treatment effect in comparison with control group)

^a^counts based on ticks attached to the dogs, excluding ticks in the crates

In the NexGard™-treated group the mean tick numbers were significantly lower compared to the Control group only at the 6 h time point on Day 42 (*F*_(3,28)_ = 32.17, *P* = 0.0366) and at the 12 h point on Day 30 (*F*_(3,28)_ = 14.56, *P* = 0.0288). In the Bravecto™-treated group mean tick counts were significantly lower compared to the Control group only at the 12 h time points from Day 30 through to Day 56 (*F*_(3, 28)_ ranged between 14.56 and 22.62 with *P*-values ranging between 0.0007 and 0.0402).

Mean tick numbers were significantly lower on all assessment days when comparing the Advantix^®^-treated group to the Control group at 3 h, 6 h and 12 h.

### Speed of kill efficacy

**Fig. 3:**
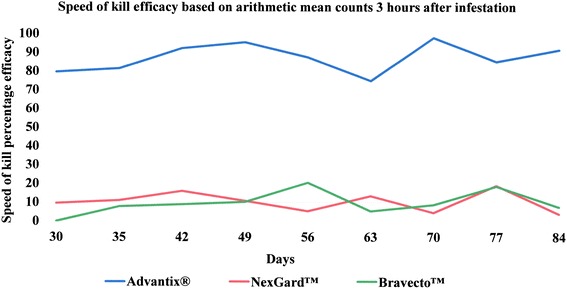
Speed of kill efficacy against *Rhipicephalus sanguineus* ticks on dogs 3 h after infestation

**Fig. 4:**
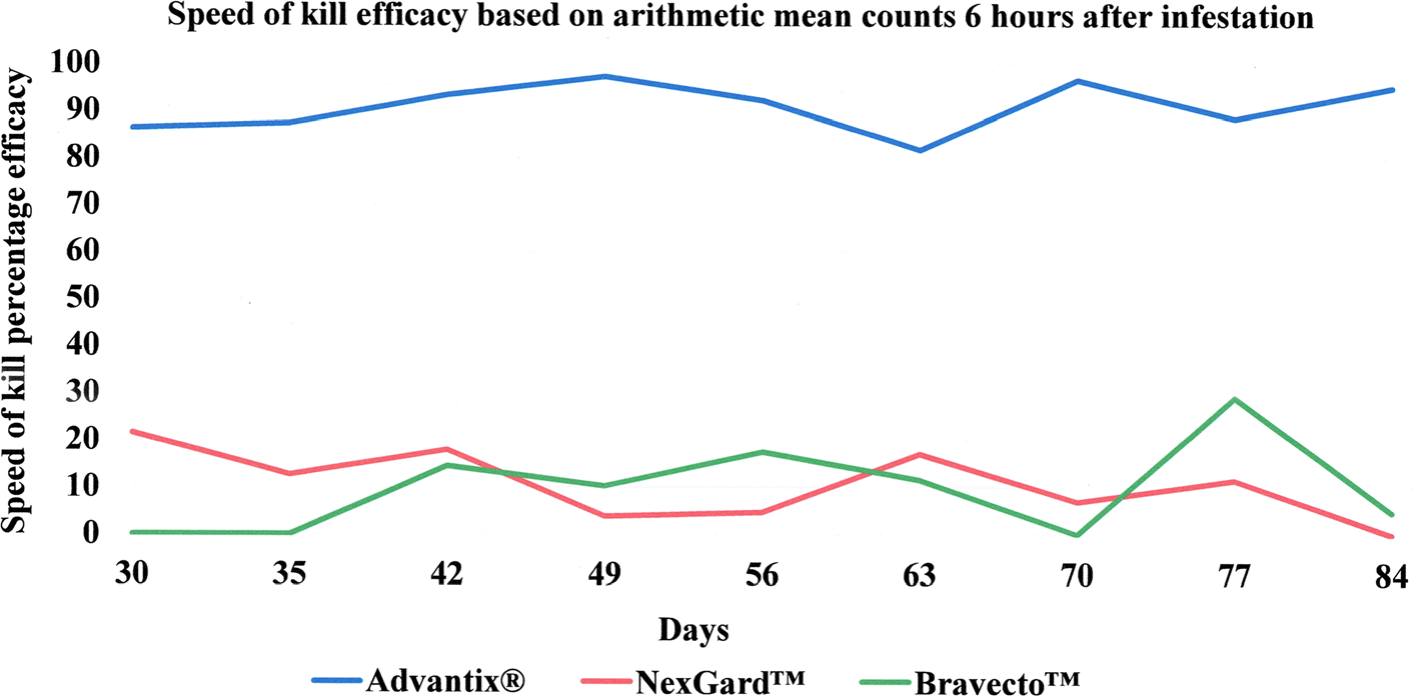
Speed of kill efficacy against *Rhipicephalus sanguineus* ticks on dogs 6 h after infestation

**Fig. 5:**
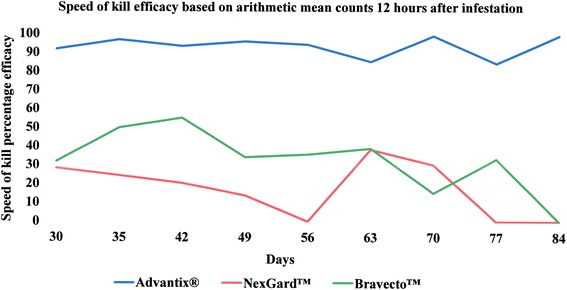
Speed of kill efficacy against *Rhipicephalus sanguineus* ticks on dogs 12 h after infestation

The comparative speed of kill induced by each of the three ectoparasiticides is illustrated in Fig. 3 for 3 h, Fig. 4 for 6 h and Fig. 5 for 12 h post-infestation, respectively. Speed of kill efficacy for the NexGard™-treated group ranged from 0 % (at the 6 h time point on Day 84 and 12 h time points on Days 56, 77 and 84) to 38.4 % (at the 12 h time point on Day 63) and exceeded 30 % on only two occasions, namely at the 12 h point on Days 63 and 70. Speed of kill efficacies for the Bravecto™-treated group ranged from 0 % (at the 3 h time point on Day 30, the 6 h time points on Days 30, 35 and 70 and the 12 h time point on Day 84) to 55.2 % (at the 12 time point on Day 42) which was also the only time point where efficacy exceeded 50 %. In the Advantix®-treated group the speed of kill efficacy ranged from 79.6 % (at the 3 h time point on Day 30) to 99.2 % (at the 12 h time point on Day 84) and exceeded 80 % at all assessment points with the exception of the 3-h point on Day 30.

### Immediate drop-off rate

The immediate drop-off rate (Table 4) assessed 3 h after infestation for the NexGard™ and Bravecto™-treated groups ranged from 0 % (both groups with time points defined in next sentence) to 7.8 and 3.8 % (both on Day 77), respectively. The NexGard™ group presented with 0 % efficacy on Days 30, 35, 42, 56, 63, 70 and 84, whilst the Bravecto™ the group presented with 0 % efficacy on Days 30, 42, 49, 63 and 70. The immediate drop-off rate for the Advantix^®^-treated group ranged from 58.3 % (on Day 63) to 77.7 % (on Day 42).

**Table 4:** Immediate drop off rate (expressed as a percentage based on arithmetic means) three hours after tick infestation

Day	Advantix^®^	NexGard™	Bravecto™
Day 30	68.4	0	0
Day 35	69.4	0	0.5
Day 42	77.7	0	0
Day 49	74.7	1.3	0
Day 56	64.6	0	0.5
Day 63	58.3	0	0
Day 70	75.7	0	0
Day 77	70.6	7.8	3.8
Day 84	75.3	0.0	0.8

### Anti-attachment efficacy

For the NexGard™-treated group anti-attachment efficacy ranged from 0 % (at the 6 and 12 h time point on Day 84) to 39.5 % (at 12 h on Day 63). For the Bravecto™-treated group anti-attachment efficacy ranged from 0 % (at the 6 h time point on Day 30 and the 12 h time point on Day 84) to 56.3 % at 12 h on Day 42. In the Advantix^®^-treated group the anti-attachment efficacy ranged between 79.8 % at the 12 h time point on Day 63 and 99.2 % at the 12 h time point on Day 84 (Tables 5 and 6).

**Table 5:** Anti-attachment efficacy (based on arithmetic means) six hours after tick infestation

Day	Advantix^®^	NexGard™	Bravecto™
Day 30	83.9	23.0	0
Day 35	90.4	22.1	23.2
Day 42	92.9	22.5	15.8
Day 49	96.8	2.1	12.0
Day 56	92.1	12.4	21.7
Day 63	80.5	19.5	15.2
Day 70	97.7	10.9	15.4
Day 77	89.7	8.3	28.9
Day 84	94.6	0.0	3.9

**Table 6:** Anti-attachment efficacy (based on arithmetic and geometric means) 12 hours after tick infestation

Day	Advantix^®^	NexGard™	Bravecto™
Day 30	90.1	31.9	36.2
Day 35	97.2	19.3	48.8
Day 42	93.7	15.1	56.3
Day 49	95.7	4.0	29.7
Day 56	94.4	0	34.6
Day 63	79.8	39.5	31.6
Day 70	99.1	24.4	5.6
Day 77	84.3	2.5	22.8
Day 84	99.2	0	0

### *Ehrlichia canis* blocking efficacy

In general, clinical signs, fever and reduced platelet counts, observed in dogs enrolled in the studies could be linked to the tick-transmitted infections (Table 7). All dogs diagnosed with *E. canis* were pyretic, except for three dogs in the Control group and one dog in the NexGard™-treated group. However, in some instances in all four groups, elevated temperature (> 39.4 °C) did not result in a confirmed diagnosis with *E. canis* and were attributed to animals being excited. Also, not all animals diagnosed infected with *E. canis* were thrombocytopenic (platelet counts < 200). Thrombocytopenia was detected in four out of six infected animals in the Control group, three out of four infected animals in the NexGard™-treated group and both infected animals in the Bravecto™-treated group.

**Table 7:** Monitoring of *Ehrlichia canis* transmission by infected *Rhipicephalus sanguineus* ticks to dogs

Group	Animal ID	Temp^a^	Platelets^b^	PCR	IFA
(1) Control	2A7 53C	40.1	199	NEG	NEG
	2AA 200	39.3	114	POS	POS
	4C6 76B	39.9	226	POS	POS
	4C6 FCC	40.5	155	POS	POS
	500 362	38.9	257	POS	POS
	CC1 5A3	40.6	172	POS	POS
	CD0 4 F5	38.8	204	NEG	NEG
	DF7 4DB	39.3	132	POS	POS
(2) Advantix^®^	284 012	38.8	392	NEG	NEG
	2AA 66 F	39.5	251	NEG	NEG
	2 AD 36C	40.3	347	NEG	NEG
	4C7 17D	40.6	396	NEG	NEG
	4DD 2E5	40.0	252	NEG	NEG
	4 F4 A1D	39.4	323	NEG	NEG
	B2B 7D0	40.4	13	NEG	NEG
	CD3 EC5	39.6	218	NEG	NEG
(3) NexGard™	289 BB6	40.6	264	NEG	NEG
	2 AC 905	40.5	70	POS	POS
	4DE 28 F	39.9	145	POS	POS
	4EF 726	40.1	27	POS	POS
	B2B 745	39.9	314	NEG	NEG
	CBF E82	39.0	231	POS	POS
	DF7 5C1	39.9	320	NEG	NEG
	E17 7C8	39.3	248	NEG	NEG
(4) Bravecto™	285 8A5	40.1	356	NEG	NEG
	2A8 C98	40.8	103	POS	POS
	4D9 0DB	40.3	134	POS	POS
	4 F7 4B1	39.6	362	NEG	NEG
	5D3 A6A	39.0	225	NEG	NEG
	E16 E41	39.0	227	NEG	NEG
	E17 4A6	39.3	184	NEG	NEG
	E18 950	39.4	202	NEG	NEG

^a^Maximum body temperature measured (> 39.4 °C is significant)

^b^Minimum platelet counts as a measure for thrombocytopenia (normal range between 200 × 10^9^/l and 500 × 10^9^/l)

*Abbreviations*: *POS*, Positive, *NEG*, Negative

Six out of eight dogs in the Control group became positive for *E. canis* DNA based on PCR analysis, indicating a successful infection challenge and consequently seroconverted. Four animals in the NexGard™-treated group and two animals in the Bravecto™-treated group tested positive for *E. canis* DNA and all of these animals also seroconverted. None of the animals in the Advantix^®^-treated group were positive for *E. canis* DNA and neither became positive for *E. canis* antibodies based on IFA analysis (Table 7).

**Fig. 6:**
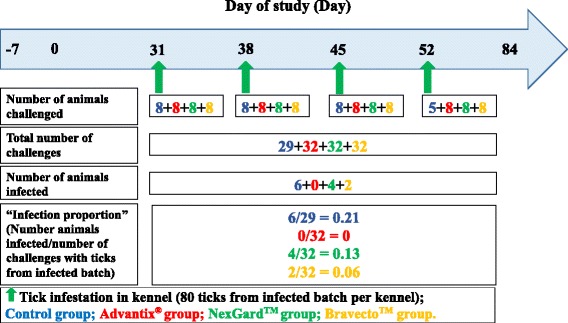
Diagrammatic representation of *Ehrlichia canis* challenge time point layout and number of animals infected per group number

The challenge periods and number of animals successfully infected are graphically presented in Fig. 6. The total number of successful challenges with ticks from an *E. canis* infected batch were 29 for the Control group and 32 for each of the respective treated groups (Advantix^®^, NexGard™ and Bravecto™. Fewer challenges were performed for the Control group as three animals were confirmed positive for *E. canis* DNA per PCR on Day 49, hence rescue-treated, and not challenged again on Day 52.

The blocking efficacy determined was 33.3 % for NexGard™ and 66.7 % for Bravecto™. Advantix^®^ fully blocked (100 %) transmission of *E. canis* to dogs over the challenge period (Table 8).

**Table 8:** *Ehrlichia canis* transmission blocking efficacy

Number infected dogs (% infected)				Efficacy (%)		
Control	Advantix^®^	NexGard™	Bravecto™	Control versus Advantix®	Control versus NexGard™	Control versus Bravecto™
6/8 (75.0)	0/8 (0)	4/8 (50.0)	2/8 (25.0)	100.0	33.3	66.7

Calculating the protection conferred against infective tick challenges, (Fig. 6) [23], NexGard™ provided 39.6 % and Bravecto™ 69.8 % protection against transmission of *E. canis* to dogs compared to 100 % protection evaluated for Advantix^®^ (Table 9).

**Table 9:** *Ehrlichia canis* protection efficacy

Number infected (proportion)				Efficacy (%)		
Control	Advantix^®^	NexGard™	Bravecto™	Control versus Advantix®	Control versus NexGard™	Control versus Bravecto™
6/29 (0.2068966)	0/32 (0)	4/32 (0.125)	2/32 (0.0625)	100.0	39.6	69.8

## Discussion

In this study we assessed the capacity to block transmission of *E. canis* by infected *R. sanguineus* ticks to dogs for two systemic compounds: afoxolaner (NexGard™, Merial Limited) [26] and fluralaner (Bravecto™, Intervet International) [27] in comparison to a reference topical 50 % permethrin/10 % imidacloprid spot-on formulation (Advantix^®^, Bayer Animal Health), which had previously shown its ability to block transmission of *E. canis* in the first month of application [14]. The speed of kill, immediate drop-off rate and anti-attachment efficacy of the three products were also studied.

The study lay out over a timeframe of three months (84 days) was owed to the fact that it compared two products licensed for monthly use (NexGard™, Advantix^®^) against a 3 months product (Bravecto™ that, however, for *R. sanguineus* has a two months label only in the EU. The challenge period for *E. canis* blocking in month 2 of the study, was chosen to include the end of the licensed efficacy period (according to the EU label) (Days 28 to 56, with first environmental tick challenge on Day 31 and last environmental tick challenge on Day 52), while challenges for tick efficacy commenced until the end of month 3 (Days 28 to 84, with first infestation for tick efficacy assessments with subsequent counts on Day 30 and last on Day 84). Advantix^®^ was used as a reference product as its ability to block the transmission of *E. canis* in the first month after application was previously shown [16]. This earlier study showed the four week efficacy against *R. sanguineus* ticks to range between 96.1 and 98.9 % at 48 h post tick challenge. Four out of six control dogs developed clinical signs of CME and required treatment, whereas none of the treated dogs became infected with *E. canis*, resulting in a blocking efficacy of 100 % [16].

In the current study on animal tick counts in the Advantix^®^-treated group were significantly lower throughout the study compared to counts on negative control dogs (Tables 1, 2 and 3). In contrast, on animal tick counts in the groups treated systemically (NexGard™, Bravecto™ were not significantly lower compared to the counts in the control group with the exception of few singular time points.

The protective ability of a topically applied acaricide with topical efficacy is due to its efficacy on contact, prior to potential attachment and feeding [16]. As for systemic acaricides the ticks need to attach to the treated host and start to take a blood meal before being killed in the process, the protection afforded is less obvious. Therefore, in the light of risk reduction for transmission of disease pathogens it needs to be evaluated specifically regarding its onset of efficacy.

The high drop-off rate within 3 h seen for Advantix^®^ due to its permethrin component was not expected to be similar in the groups treated with the systemic compounds, which was confirmed by the results (Table 4).

Similarly, the subsequent anti-attachment efficacy, which evaluated if ticks remaining on the animals at 3 h after infestation actually attached to the animals by 6 h and 12 h, respectively, was lower in the orally treated groups (NexGard™, Bravecto™) than in the Advantix^®^-treated group, concurring with the need of ticks to attach and take a blood meal to be exposed to the systemically acting actives.

Speed of kill efficacies at 3 h, 6 h and 12 h after tick infestation, showed the efficacy for the NexGard™-treated group to exceeded 30 % only at the 12 h time points on Days 63 and 70 (Table 6). Likewise, speed of kill efficacies for the Bravecto™-treated group exceeded 50 % only on a single occasion at 12 h on Day 42 (Table 6). 0 % efficacy was measured for both oral products at the 12 h point on Day 84 at the end of the study the efficacy (Table 6). This means that tick count numbers were not significantly different any longer from the level encountered on dogs of the negative control group (Table 3). With respect to speed of kill and resulting onset of acaricidal efficacy, Advantix^®^ was superior over NexGard™ and Bravecto™ at all time points (3 h, 6 h and 12 h) in the 12 h period observed (Figs. 1, 2 and 6).

The high variability in efficacy seen at these early assessment time points is assumed to depend largely on the actual time point at which the ticks attach and commence feeding after infestations. This differs for topically applied products with topical efficacy, which start to work on contact, thus directly at infestation and prior to potential attachment and feeding [16].

The speed of kill efficacies observed in our study for NexGard™ and Bravecto™ against *R. sanguineus* are slightly lower than those obtained in earlier publications for early time points, however, follow the same trend regarding variability. Varloud et al. (2015) found speed of kill efficacies (based on geometric means) 12 h after infestation ranging between 21–49 % for NexGard™ and 58–89 % for Bravecto™ [24]. Ohmes et al. (2015) found speed of kill efficacies (based on geometric means) for NexGard™ ranging between 0–26 % 3 h after infestation and 12–45 % 12 h after infestation and for Bravecto™ ranging between 13–26 % 3 h after infestation and 52–98 % 12 h after infestation [25].

Similarly to the higher variability seen in the efficacy of systemic acaricides at early time points, it is also expected to see a higher variability in the efficacy between different tick species due to differences in attachment and feeding behaviour [16]. For instance, the immediate speed of kill efficacy published for NexGard™ against *Ixodes ricinus* was reported to reach 93.4 % within 12 h post treatment with the persistent speed of kill at the 12 h time point ranging between 76.6 % on Day 7 and 38.5 % on Day 28 [33]. For Bravecto™ the speed of kill efficacy against existing infections (immediate speed of kill) with *I. ricinus* was found to be 89.6 % at 4 h, 97.9 % at 8 h and 100 % at 12 h after treatment. The speed of kill efficacy against reinfections (persistent speed of kill) against *I. ricinus* was found to range from 33.2 % in week four after treatment to 7.8 % in week 12 after treatment at 4 h, from 96.8 % in week four after treatment to 45.8 % in week 12 after treatment at 8 h and from 99.7 % in week four after treatment to 98.3 % in week 12 after treatment at 12 h [34].

However speed of kill efficacies are ultimately only an indicator for the potential to block transmission of disease pathogens. A key issue with respect to any acaricidal product is its capacity to prevent infection by blocking transmission of pathogens that can be transmitted by ticks and may cause disease in companion animals. This capacity depends on the time it takes the product to be fully effective, but also on the time interval before the tick is able to transmit the pathogen [16].

This is the first study wherein both afoxolaner as well as fluralaner have been tested for their ability to prevent transmission of *E. canis*. The *E. canis* infected *R. sanguineus* tick batch used for challenges in this study had an average infection rate of 3.8 %. Prevalence reported for *E. canis* in *R. sanguineus* ticks from field collections range between 0.09–27 % [35–38], varying widely between different endemic areas. The use of 80 ticks per challenge resulted in an adequate infection, as 6 out of the 8 control animals got infected.

The capacity of the two systemic compounds to block transmission of *E. canis* by infected *R. sanguineus* ticks to dogs over the challenge period was assessed to be 33.3 % for NexGard™ and 66.7 % for Bravecto™. Advantix^®^ effectively blocked transmission of *E. canis* to dogs by 100 % for the challenge period (Table 8), thus reconfirming the result observed in an earlier study for the first month after application [14]. Clearly, the speed of kill of ticks of NexGard™ was not sufficiently fast to prevent transmission of *E. canis* in 4 out of 32 infected tick challenges, whereas in 2 out of 32 challenges transmission was not prevented by Bravecto™. Thus the protection results conferred against infective tick challenges (Fig. 6) were 39.6 % for NexGard™ and 69.8 % for Bravecto™, while Advantix^®^ provided 100 % protection against *E. canis* to dogs over the challenge period (Table 9). Comparing results from both the blocking and protection calculation methods employed, results were found to be in the same order. However, a slightly higher protection percentage was calculated and is considered more representative as it also considers the number of challenges with the infected tick strain.

Although the published persistent speed of kill of NexGard™ [33] and Bravecto™ [34] is sufficient to prevent transmission of the slower transmitted pathogen *B. canis*, in this study for the quick transmitted *E. canis* we were only able to demonstrate low partial blocking and protection capacity. It is generally accepted that protozoan parasites, such as *Babesia* spp., require additional time for their sporoblasts to mature into infective sporozoites in the acini of the salivary glands of the vector ticks. Two studies have been published wherein it was shown that both systemic compounds effectively blocked transmission of *B. canis* by *D. reticulatus* ticks. The ability of NexGard™ to block transmission of *B. canis* was determined in two groups of 8 dogs [19]. It was found that all treated dogs remained negative for a period of 28 days, whereas all untreated control required treatment for babesiosis [19]. In a similar study design, prevention of transmission of *B. canis* by *D. reticulatus* ticks to dogs orally treated with Bravecto™ was also demonstrated. Eight control dogs became infected with *B. canis*, but none of the eight dogs treated with Bravecto™ over a period of 12 weeks [20].

Finally, looking at the differences in transmission time for the different tick-borne pathogens from the perspective of risk minimization for pathogen transmission, accounting for the “worst case scenario” of a quickly transmitted pathogen is considered to be a more accurate scenario for the evaluation of a general risk prevention potential.

## Conclusions

Dogs treated with the Advantix^®^ spot-on carried significantly less *R. sanguineus* ticks throughout the study as compared with the negative controls and was with respect to the speed of kill and resulting onset of the acaricidal efficacy superior over NexGard™ and Bravecto™ at all time points (3 h, 6 h and 12 h) in the 12 h period observed.

The speed of kill of the both systemic compounds against *R. sanguineus* was not sufficiently fast to prevent transmission of *E. canis* and resulted in only partial blocking and protection capacity while Advantix^®^ effectively blocked transmission of *E. canis* to dogs in the challenge period and thus provided adequate protection for dogs against monocytic ehrlichiosis.

## Abbreviations

ANOVA, analysis of variance; CBC, complete blood count; CME, canine monocytic ehrlichiosis; Day, study day; DNA, deoxyribonucleic acid; Fig, Figure; GCP, Good Clinical Practice; h, hour; IFA, immunofluorescence assay; kg, kilogram; mg, milligram; min, minute; PCR, polymerase chain reaction; TBDs, tick-borne diseases; TBE, tick-borne encephalitis; UCTD, utrecht centre for tick-borne diseases; VBDs, vector-borne diseases; VICH, Veterinary International Conference on Harmonization.

## References

[1] Jongejan F, Uilenberg G (2004). The global importance of ticks. Parasitology.

[2] Walker JB, Keirans JE, Horak IG. The genus Rhipicephalus (Acari:Ixodidae): a guide to the brown Ticks of the world. Cambridge, UK: Cambridge University Press; 2000.

[3] Nava S, Estrada-Peña A, Petney T, Beati L, Labruna MB, Szabó MPJ, Venzal JM, Mastropaolo M, Mangold AJ, Guglielmone AA (2015). The taxonomic status of *Rhipicephalus sanguineus* (Latreille, 1806). Vet Parasitol..

[4] Harrus S, Waner T (2011). Diagnosis of canine monocytotropic ehrlichiosis (*Ehrlichia canis*): an overview. Vet J..

[5] René-Martellet M, Lebert I, Chêne J, Massot R, Leon M, Leal A, Badavelli S, Chalvet-Monfray K, Ducrot C, Abrial D, Chabanne L, Halos L (2015). Diagnosis and incidence risk of clinical canine monocytic ehrlichiosis under field conditions in Southern Europe. Parasit Vectors..

[6] Sainz Á, Roura X, Miró G, Estrada-Peña A, Kohn B, Harrus S, Solano-Gallego L (2015). Guideline for veterinary practitioners on canine ehrlichiosis and anaplasmosis in Europe. Parasit Vectors..

[7] Fourie JJ, Horak I, Crafford D, Erasmus HL, Botha OJ (2015). The efficacy of a generic doxycycline tablet in the treatment of canine monocytic ehrlichiosis. J S Afr Vet Assoc..

[8] Dantas-Torres F (2010). Biology and ecology of the brown dog tick, *Rhipicephalus sanguineus*. Parasit Vectors.

[9] Demma LJ, Traeger MS, Nicholson WL, Paddock CD, Blau DM, Eremeeva ME, Dasch GA, Levin ML, Singleton J, Zaki SR, Cheek JE, Swerdlow DL, McQuiston JH (2005). Rocky Mountain spotted fever from an unexpected tick vector in Arizona. N Engl J Med..

[10] Dantas-Torres F (2008). The brown dog tick, *Rhipicephalus sanguineus* (Latreille, 1806) (Acari: Ixodidae): from taxonomy to control. Vet Parasitol..

[11] Alekseev AN, Chunikhin SP (1990). The experimental transmission of the tick-borne encephalitis virus by ixodid ticks (the mechanisms, time periods, species and sex differences). Parazitologiia..

[12] Schein E, Mehlhorn H, Voigt WP (1979). Electron microscopical studies on the development of *Babesia canis* (Sporozoa) in the salivary glands of the vector tick *Dermacentor reticulatus*. Acta Trop..

[13] Nicholson WL, Allen KE, McQuiston JH, Breitschwerdt EB, Little SE (2010). The increasing recognition of rickettsial pathogens in dogs and people. Trends Parasitol..

[14] Fourie JJ, Luus HG, Stanneck D, Jongejan F (2013). The efficacy of Advantix® to prevent transmission of *Ehrlichia canis* to dogs by *Rhipicephalus sanguineus* ticks. Parasite..

[15] Saraiva DG, Soares HS, Soares JF, Labruna MB (2014). Feeding period required by *Amblyomma aureolatum* ticks for transmission of *Rickettsia rickettsii* to vertebrate hosts. Emerg Infect Dis..

[16] Fourie JJ. Integrated control of ticks and fleas on dogs with particular reference to the prevention of vector-borne diseases. PhD thesis, Utrecht University, Utrecht, The Netherlands. 2015; 1-180. ISDN 978-90-393-6407-9.

[17] Jongejan F, Fourie JJ, Chester ST, Manavella C, Mallouk Y, Pollmeier MG, Baggott D (2011). The prevention of transmission of *Babesia canis canis* by *Dermacentor reticulatus* ticks to dogs using a novel combination of fipronil, amitraz and (S)-methoprene. Vet Parasitol..

[18] Fourie JJ, Stanneck D, Jongejan F (2013). Prevention of transmission of *Babesia canis* by *Dermacentor reticulatus* ticks to dogs treated with an imidacloprid/flumethrin collar. Vet Parasitol..

[19] Beugnet F, Halos L, Larsen D, Labuschagné M, Erasmus H, Fourie J (2014). The ability of an oral formulation of afoxolaner to block the transmission of *Babesia canis* by *Dermacentor reticulatus* ticks to dogs. Parasit Vectors..

[20] Taenzler J, Liebenberg J, Roepke RKA, Heckeroth AR (2015). Prevention of transmission of Babesia canis by *Dermacentor reticulatus* ticks to dogs treated orally with fluralaner chewable tablets (Bravecto™. Parasit Vectors..

[21] Fourie JJ, Ollagnier C, Beugnet F, Luus HG, Jongejan F (2013). Prevention of transmission of *Ehrlichia canis* by *Rhipicephalus sanguineus* ticks to dogs treated with a combination of fipronil, amitraz and (S)-methoprene (Certifect®). Vet Parasitol..

[22] Stanneck D, Fourie JJ (2013). Imidacloprid 10 % / Flumethrin 4.5 % collars (Seresto®, Bayer) successfully prevent long-term transmission of *Ehrlichia canis* by infected *Rhipicephalus sanguineus* ticks to dogs. Parasitol Res.

[23] Jongejan F, de Vos C, Fourie JJ, Beugnet F (2015). A novel combination of fipronil and permethrin (Frontline Tri-Act®/Frontect®) reduces risk of transmission of *Babesia canis* by *Dermacentor reticulatus* and of *Ehrlichia canis* by *Rhipicephalus sanguineus* ticks to dogs. Parasit Vectors..

[24] Varloud M, Liebenberg J, Fourie J. Comparative speed of kill between a topical administration of dinotefuan-permethrin-pyriproxifen and an oral administration of afololaner of fluralaner to dogs weekly infested for one month with *Rhipicephalus sanguineus* ticks. In Proceedings of the 2015 AAVP, LIWC, ISEP meeting, Massachusetts, United States of America; 2015:68.

[25] Ohmes C, Hostetler J, Wendell L, Setje T, Everett W. Comparative efficacy of imidacloprid/permethrin/pyriproxyfen (K9 Advantix® II), afoxolaner (NexGard ®) and fluralaner (Bravecto®) against tick (*Rhipicephalus sanguineus* and *Amblyomma americanum*) infections on dogs. In Proceedings of the 2015 AAVP, LIWC, ISEP meeting, Massachusetts, United States of America; 2015:69.

[26] Shoop WL, Hartline EJ, Gould BR, Waddell ME, McDowell RG, Kinney JB, Lahm GP, Long JK, Xu M, Wagerle T, Jones GS, Dietrich RF, Cordova D, Schroeder ME, Rhoades DF, Benner EA, Confalone PN (2014). Discovery and mode of action of afoxolaner, a new isoxazoline parasiticide for dogs. Vet Parasitol..

[27] Gassel M, Wolf C, Noack S, Williams H, Ilg T (2014). The novel isoxazoline ectoparasiticide fluralaner: selective inhibition of arthropod γ-aminobutyric acid- and L-glutamate-gated chloride channels and insecticidal/acaricidal activity. Insect Biochem Mol Biol..

[28] VICH GL9 Good clinical practice. 2000. Implemented July 2001. International Cooperation on Harmonisation of Technical Requirements for Registration of Veterinary Medicinal Products, Bruxelles, Belgium.

[29] EMEA/CVMP/005/2000-Rev.2. Guideline for the testing and evaluation of the efficacy of antiparasitic substances for the treatment and prevention of tick and flea infestation in dogs and Cats. 2007. Committee for Veterinary Medicinal Product of the European Agency for the Evaluation of Medicinal Products, London, United Kingdom.

[30] Marchiondo AA, Holdsworth PA, Fourie LJ, Rugg D, Hellmann K, Snyder DE, Dryden MW (2013). World Association for the Advancement of Veterinary Parasitology (W.A.A.V.P.) second edition: guidelines for evaluating the efficacy of parasiticides for the treatment, prevention and control of flea and tick infestations on dogs and cats. Vet Parasitol.

[31] Fourie JJ, Stanneck D, Luus HG, Beugnet F, Wijnveld M, Jongejan F (2013). Transmission of Ehrlichia canis by *Rhipicephalus sanguineus* ticks feeding on dogs and on artificial membranes. Vet Parasitol..

[32] Navarro C, Reymond N, Fourie J, Hellmann K, Bonneau S (2015). Prevention of *Babesia canis* in dogs: efficacy of a fixed combination of permethrin and fipronil (Effitix®) using an experimental transmission blocking model with infected *Dermacentor reticulatus* ticks. Parasit Vectors..

[33] Halos L, Lebon W, Chalvet-Monfray K, Larsen D, Beugnet F (2014). Immediate efficacy and persistent speed of kill of a novel oral formulation of afoxolaner (NexGard™ against induced infestations with *Ixodes ricinus* ticks. Parasit Vectors..

[34] Wengenmayer C, Williams H, Zschiesche E, Moritz A, Langenstein J, Roepke RK, Heckeroth AR (2014). The speed of kill of fluralaner (Bravecto™ against *Ixodes ricinus* ticks on dogs. Parasit Vectors..

[35] Araes-Santos AI, Moraes-Filho J, Peixoto RM, Spolidorio MG, Azevedo SS, Costa MM, Labruna MB, Horta MC (2015). Ectoparasite infestations and canine infection by rickettsiae and ehrlichiae in a semi-arid region of northeastern Brazil. Vector Borne Zoonotic Dis..

[36] Socolovschi C, Gomez J, Marié J-L, Davoust B, Guigal P-M, Raoult D, Parola P (2012). *Ehrlichia canis* in *Rhipicephalus sanguineus* ticks in the Ivory Coast. Ticks Tick Borne Dis.

[37] Aguiar DM, Cavalcante GT, Pinter A, Gennari SM, Camargo LMA, Labruna MB (2007). Prevalence of *Ehrlichia canis* (Rickettsiales: Anaplasmataceae) in dogs and *Rhipicephalus sanguineus* (Acari: Ixodidae) ticks from Brazil. J Med Entomol..

[38] Satta G, Chisu V, Cabras P, Fois F, Masala G (2011). Pathogens and symbionts in ticks: a survey on tick species distribution and presence of tick-transmitted micro-organisms in Sardinia. Italy. J Med Microbiol..

